# TSG (2,3,5,4′-Tetrahydroxystilbene-2-O-***β***-D-glucoside) from the Chinese Herb *Polygonum multiflorum* Increases Life Span and Stress Resistance of *Caenorhabditis elegans*

**DOI:** 10.1155/2015/124357

**Published:** 2015-05-05

**Authors:** Christian Büchter, Liang Zhao, Susannah Havermann, Sebastian Honnen, Gerhard Fritz, Peter Proksch, Wim Wätjen

**Affiliations:** ^1^Institute of Agricultural and Nutritional Sciences, Martin-Luther-University Halle-Wittenberg, Weinbergweg 22, 06120 Halle/Saale, Germany; ^2^Institute of Toxicology, Heinrich-Heine-University, Moorenstrasse 5, 40225 Düsseldorf, Germany; ^3^Institute of Pharmaceutical Biology and Biotechnology, Heinrich-Heine-University, Universitätsstraße 1, 40225 Düsseldorf, Germany

## Abstract

2,3,5,4′-Tetrahydroxystilbene-2-O-*β*-D-glucoside (TSG) was isolated from *Polygonum multiflorum*, a plant which is traditionally used as an anti-ageing drug. We have analysed ageing-related effects of TSG in the model organism *C. elegans* in comparison to resveratrol. TSG exerted a high antioxidative capacity both in a cell-free assay and in the nematode. The antioxidative capacity was even higher compared to resveratrol. Presumably due to its antioxidative effects, treatment with TSG decreased the juglone-mediated induction of the antioxidative enzyme SOD-3; the induction of the GST-4 by juglone was diminished slightly. TSG increased the resistance of *C. elegans* against lethal thermal stress more prominently than resveratrol (50 *μ*M TSG increased mean survival by 22.2%). The level of the ageing pigment lipofuscin was decreased after incubation with the compound. TSG prolongs the mean, median, and maximum adult life span of *C. elegans* by 23.5%, 29.4%, and 7.2%, respectively, comparable to the effects of resveratrol. TSG-mediated extension of life span was not abolished in a DAF-16 loss-of-function mutant strain showing that this ageing-related transcription factor is not involved in the effects of TSG. Our data show that TSG possesses a potent antioxidative capacity, enhances the stress resistance, and increases the life span of the nematode *C. elegans*.

## 1. Introduction

Ageing is defined as an accumulation of diverse deleterious changes occurring in cells and tissues with advancing age that are responsible for the increased risk of several diseases and finally death [1]. Oxidative stress is believed to play a role in both physiological and pathological ageing processes, for example, age-related neurodegenerative diseases. However, besides the free radical theory of ageing [2], several other theories exist, for example, the hyperfunction theory of ageing [3], the inflammation theory of ageing [4], and the mitochondrial theory of ageing [5], which shows the complexity of the ageing process. A number of herbal medicines have been used traditionally to increase longevity and health. For example, extracts of* Polygonum multiflorum* are used as an anti-ageing treatment in East Asian countries. Besides the traditional use in folk medicine, extracts of* Polygonum multiflorum* have been shown to possess anti-ageing effects in different species: Chan et al. [6] reported that mice fed with* Polygonum multiflorum* extract had less lipofuscin in the hippocampus and lower MDA concentrations in the brain. An extract consisting of* Polygonum multiflorum* reduced the lipofuscin content of liver and brain tissues in both young (1 month old) and adult (11 months old) mice [7]. Li et al. [8] showed neuroprotective effects of an extract on nigrostriatal degeneration in mice.* Polygonum multiflorum* (root) was able to lower A*β* generation by modulating APP processing* in vitro* [9] and to prevent A*β*-induced increase of thiobarbituric acid reactive substances and cognitive deficits in mice [10]. Steele et al. [11] reported cytoprotective effects of an extract of* Polygonum multiflorum* in astroglia cells. Furthermore, an improvement of cognitive performance in senescence accelerated mice [12] and an attenuation of glutamate-induced neurotoxicity [13] were demonstrated. Since many of the constituents of herbal extracts possess an antioxidative capacity, it is believed that this property may be involved, at least in parts, in the anti-ageing mechanism of the herbal extract. The stilbene glucoside 2,3,5,4′-tetrahydroxystilbene-2-O-*β*-D-glucoside (TSG) is the main bioactive component of* Polygonum multiflorum* [14]; other compounds isolated were physcion, apigenin, hyperoside, rutin, vitexin, beta-amyrin, beta-sitosterol, and daucosterol. TSG is a potent antioxidant: Chen et al. [15] and Ryu et al. [16] investigated the antioxidant activity of compounds isolated from* Polygonum multiflorum* (DPPH-assay) showing TSG as an active constituent. TSG exerts protective effects on experimental colitis through diminishing the level of oxygen and nitrogen free radicals [17]. Wang et al. [18] showed that TSG has protective effects against cerebral ischemia by modulation of JNK, SIRT1, and NF-*κ*B pathways. TSG mediates antagonistic effects on oxidation of lipoprotein, proliferation, and decrease of NO content of coronary arterial smooth muscle cells, which partially explains the antiatherosclerosis mechanism of* Polygonum multiflorum* [19]. Lv et al. [20] reported that TSG efficiently inhibits the formation of advanced glycation end products (AGEs). TSG has been shown to exert positive effects on learning and memory in animal models. This compound was shown to be protective against apoptosis in rat adrenal pheochromocytoma cells by modulating the ROS-NO pathway [21]. Sun et al. [22] showed that TSG protects human neuroblastoma SH-SY5Y cells against MPP^+^-induced cell death through improving mitochondrial function, decreasing oxidative stress and inhibiting apoptosis which may be relevant for treatment of Parkinson's disease. Protective effects of TSG against MPP^+^-toxicity in PC12 cells were reported to be mediated via modulation of the phosphoinositide-3-kinase/Akt signaling pathway [23] and the JNK pathway [24]. Various studies were performed concerning anti-ageing effects of TSG; however up to now, no direct correlation between ageing and TSG has been published. To investigate the effects of TSG on ageing processes, we used the model organism* Caenorhabditis elegans*. It is known that several polyphenols, for example, quercetin [25], myricetin [26], catechin [27], epigallocatechin gallate [28], or baicalein [29], increase the life span of* C. elegans*. As molecular mechanism for the life span extension, modulation of the insulin/IGF-like signalling pathway or an activation of Sir2 (sir-2.1 in* C. elegans*), a member of the sirtuin family of NAD^+^-dependent deacetylases is discussed. Resveratrol acts on a number of target proteins [30]; for example, it was recently shown to activate AMP-kinase and to exert neuroprotective properties independent of Sir2 [31].

*Aim of the Study. Polygonum multiflorum* is widely used as a traditional anti-ageing drug in East Asian countries. The main bioactive component of the extract is 2,3,5,4′-tetrahydroxystilbene-2-O-*β*-D-glucoside (TSG). The aim of this study was to evaluate the effects of TSG on oxidative stress, stress resistance, and life span in the model organism* C. elegans* in comparison to the structural analogue resveratrol and the flavonoid quercetin. For this reason, antioxidative effects were investigated* in vitro* and* in vivo*; effects on stress resistance, lipofuscin accumulation and expression of protective enzymes, and the life span were investigated.

## 2. Materials and Methods

### 2.1. Materials

Resveratrol and trolox were purchased from Calbiochem (Merck, Darmstadt, Germany), quercetin was from Extrasynthese (Genay, France), and DMSO was from Merck (Darmstadt, Germany). All other chemicals were of analytical grade and were purchased from Sigma (Deisenhofen, Germany).

### 2.2. Isolation of 2,3,5,4′-Tetrahydroxystilbene-2-O-*β*-D-glucoside

The Chinese medicine plant* Polygonum multiflorum* was bought from Hangzhou Zhongmei Huadong Pharmaceutical Co., Ltd., Hangzhou, China. Isolation and purification of 2,3,5,4′-tetrahydroxystilbene-2-O-*β*-D-glucoside were performed at the Heinrich-Heine-University, Institute of Pharmaceutical Biology and Biotechnology.

### 2.3. *C. elegans* Strains and Maintenance

The strains used in this study were N2 var. Bristol, CF1038* [daf-16(mu86) I.]*, CF1553* [muIs84(sod-3p::gfp)]*, and CL2166* [pAF15(gst-4p::GFP::NLS)]*. All strains were provided by the* Caenorhabditis* Genetics Center (CGC), which is funded by NIH Office of Research Infrastructure Programs (P40 OD010440). Strains were maintained on nematode growth medium (NGM) agar plates at 20°C containing a lawn of* E. coli* var. OP50 (provided by the CGC) as the food source, as described previously [32]. Treatment of* C. elegans* with the substances was performed in 2 mL of liquid NGM containing 1% (w/v) bovine serum albumin, 50 *μ*g/mL streptomycin, and 10^9^ OP50-1/mL (provided by the CGC) in 35 mm petri dishes (Greiner Bio-One, Frickenhausen, Germany). Stock solutions (100 mM) were prepared with DMSO. In all assays, the substances were used in a final concentration of 50 or 100 *μ*M; 0.1% (v/v) DMSO was used as the solvent control. Age synchronous animals were obtained by sodium hypochlorite treatment of gravid adults. Briefly, gravid adults were rinsed off from NGM agar plates with liquid NGM, collected in 0.5 mL liquid NGM and mixed with 0.5 mL bleaching solution (50% 5 M NaOH/50% NaClO). Nematodes were then incubated at room temperature for three minutes, occasionally vortexed, pelleted by centrifugation (5000 rpm/4°C/1 min) and the supernatant was discarded. The animal pellet was washed three times with liquid NGM and transferred onto fresh NGM agar plates (containing OP50 lawn) and maintained for three days at 20°C to obtain an age synchronous population of mainly L4 larvae.

### 2.4. Determination of Antioxidative Capacity* In Vitro*

To determine the antioxidative capacity of quercetin, resveratrol, and TSG, the TEAC assay was used. This assay is a cell-free method for the measurement of radical scavenging properties of compounds [33]. The principle of this reaction is a reductive conversion of a stable, blue-green radical by an antioxidant. The solution decolorizes when an antioxidant is added and can be quantified spectrophotometrically. The decolorisation of the radical solution indicates the antioxidative capacity of a compound which is compared to the potency of the reference substance trolox, which is a synthetic vitamin E derivative. The reference- and test-substances were measured in a concentration range from 0 to 25 *μ*M. The radical scavenging activity was measured at 734 nm spectrophotometrically (Lambda 25 UV/VIS Spectrometer, Perkin Elmer, Wellesley, MA, USA) two minutes after starting the reaction.

### 2.5. Measurement of Intracellular ROS Accumulation* In Vivo*

The fluorescent probe H_2_DCF-DA (2′,7′-dichlorodihydrofluorescein-diacetate) was used to detect the intracellular ROS level in living individual nematodes. H_2_DCF-DA is able to freely cross cell membranes; however, after entering the cell, nonfluorescent H_2_DCF-DA becomes deacetylated to form the nonfluorescent derivative H_2_DCF that is trapped within the cell. Then H_2_DCF can quickly be oxidised by intracellular ROS to form fluorescent DCF that can be measured in a fluorescence spectrophotometer (excitation wavelength 485 nm; emission wavelength 535 nm). The fluorescence intensity correlates with the intracellular amount of ROS; no saturation of the DCF fluorescence was observed up to 8 h of persistent thermal stress. The experiment was performed as described elsewhere [34]. Briefly, L4 larvae were incubated in liquid NGM ± the compounds (50/100 *μ*M) or 0.1% DMSO for 48 hours at 20°C. During the incubation period, nematodes were transferred to fresh culture media daily. After 48 hours, all animals were transferred into M9T (M9 buffer containing 0.1% Tween 20) for one hour. Then single nematodes were transferred individually in 1 *μ*L M9T into each well of a 384-well plate (384-well *μ*Clear plate, Greiner Bio-One, Frickenhausen, Germany) containing 7 *μ*L M9 buffer. Subsequently, when all animals were transferred, 2 *μ*L H_2_DCF-DA (250 *μ*M in PBS) was added into each well to obtain a final concentration of 50 *μ*M H_2_DCF-DA. A black backing tape (Perkin Elmer) was applied to the top of the plate to avoid evaporation. ROS accumulation was induced by thermal stress at 37°C and recorded every 15 min for a period of 12 hours in a fluorescence spectrophotometer (Wallac Victor^2^ 1420 Multilabel-Counter, Perkin Elmer, Wellesley, MA, USA).

### 2.6. Determination of Lipofuscin Accumulation

Over the lifetime of* C. elegans*, the autofluorescent pigment lipofuscin accumulates in gut granules and serves as an established marker of ageing. Randomly picked L4 larvae were placed in liquid NGM ± the compounds (50/100 *μ*M) as described above and incubated for 72 hours at 20°C, followed by 24 hours of incubation in compound free medium. During the incubation period, nematodes were transferred to fresh culture media daily. Lipofuscin fluorescence of seven-day-old nematodes was detected by fluorescence microscopy (excitation wavelength 360–370 nm; emission wavelength 420–460 nm; Olympus BX43; Olympus, Hamburg, Germany) and analysed densitometrically (ImageJ, National Institutes of Health, Bethesda, MD, USA). The experiment was repeated three times and 20 animals per group and experiment were analysed.

### 2.7. Induction of Antioxidative Enzymes

Immediately after the bleaching procedure, synchronised eggs of the transgenic strains CF1553 (expressing a* sod-3p::gfp* reporter) or CL2166 (expressing a* gst-4p::gfp::nls* reporter) were incubated in liquid NGM containing 100 *μ*M of quercetin, resveratrol, TSG, or 0.1% DMSO, respectively, as described above and incubated for five days at 20°C. After five days of drug treatment, nematodes were placed in PBST (phosphate buffered saline containing 0.1% Tween 20) for 30 minutes to wash off residual bacteria. Each group was then separated into two groups, exposing one of each group to 150 *μ*M of the juglone for 3 hours. The naphthoquinone juglone is a redox cycler, which results in the generation of reactive oxygen species. A cyclic process consisting of (a) reduction of a compound, followed by (b) autoxidaton of the reaction product leads to a prolonged production of ROS. 10 to 15 animals from each group were placed onto microscope slides, anesthetized in 10 *μ*L of 10 mM levamisole, and covered with cover slips. Epifluorescence images were collected from an Axiolab fluorescence microscope (Zeiss, Göttingen, Germany) using a suitable filter set with a CoolSnap CF Digital Monochrome Camera (Intas, Göttingen, Germany) equipped with the Image ProPlus software (version 4.5, MediaCybernetics, Silver Spring, MD, USA). Densitometric analysis of GFP expression of the head and anterior intestinal area of at least 10 animals per group and experiment was performed using ImageJ software (National Institutes of Health, Bethesda, MD, USA).

### 2.8. Thermotolerance Assay

Survival of individual nematodes at the lethal temperature of 37°C was monitored with an assay according to Gill et al. [35] and Kampkötter et al. [34]. After treating wild type L4 larvae for 48 hours with the compounds (50/100 *μ*M) or 0.1% DMSO (daily transfer of the animals into fresh culture medium), nematodes were washed in PBST for one hour and then individually transferred in 1 *μ*L PBST into the wells of a 384-well plate (384-well *μ*Clear plate, Greiner Bio-One, Frickenhausen, Germany) containing 9 *μ*L PBS. Following the complete transfer of the nematodes, 10 *μ*L of 2 *μ*M SYTOX Green Nucleic Acid Stain (Molecular Probes Inc.; Leiden, The Netherlands) in PBS were added to each well and the plate was sealed using black backing tape (Perkin Elmer) to avoid evaporation. SYTOX Green Nucleic Acid Stain is unable to pass the membranes of intact cells. However, thermal stress causes an impairment of the cellular membrane, thereby enabling the dye to enter the cells. There the dye binds to DNA and exerts a bright fluorescence that can be used as a marker for cellular damage and thus for the viability of individual nematodes [35]. The fluorescence intensity was determined with a fluorescence spectrophotometer (Wallac Victor^2^ 1420 Multilabel-Counter, Perkin Elmer, Wellesley, MA, USA) and was recorded every 15 min for 12 hours (excitation wavelength 485 nm; emission wavelength 535 nm). The fluorescence curve of each nematode was calculated and the individual cut-off value was determined by multiplying the average fluorescence of the first four measurements by the factor 3. The time point when the fluorescence exceeded the cut-off value for each well was defined as the point of death of the respective nematode. The factor 3 in the calculation of the cut-off value was previously shown to be adequate [35]. Survival curves and mean survival times were determined from these individual times of death (Kaplan-Meier survival analysis, IBM SPSS 19). Experiments were repeated at least three times.

### 2.9. Life Span Assays

Life span analyses were performed with N2 (three independent experiments) and CF1038* [daf-16(mu86) I.]* (two independent experiments). About 30–50 L4 larvae per group and experiment were placed in liquid NGM ± compounds (50/100 *μ*M) as described above and incubated at 25°C. The starting day in liquid culture was considered as day 0 of the life span. Nematodes were transferred daily to new culture dishes during their fertile period to prevent overcrowding and to discriminate the test nematodes from their progeny. After the fertile period, nematodes were transferred to fresh medium every other day. Nematodes were scored as dead when they did not respond to gentle prodding and when they showed no pharyngeal pumping movement. Lost nematodes and animals containing hatched larvae were censored. Kaplan-Meier survival analysis was used to detect statistical differences.

### 2.10. Statistics

Data are given as mean ± S.E.M of at least 3 independent experiments. Statistical analysis was performed with SPSS 19 (SPSS Inc., Chicago, IL, USA) and GraphPad Prism 5 (GraphPad Software, Inc., LaJolla, USA) software. Statistical significance was determined by one-way ANOVA with Bonferroni posttest. Life span analyses were performed using Kaplan-Meier survival analysis; animals that were lost, killed, or showed internal hatching were censored. Differences were considered to be significant at *P* < 0.05.

## 3. Results and Discussion

### 3.1. Antioxidative Capacity of TSG

**Figure 1:**
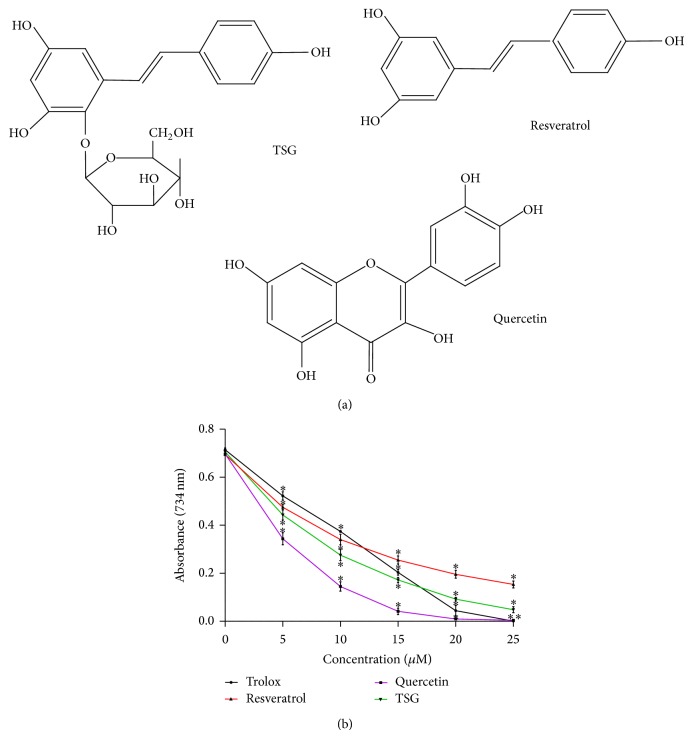
Radical scavenging properties (TEAC assay) of TSG. Chemical structures of resveratrol, quercetin, and TSG are shown in (a); the antioxidative capacity of the substances measured by the TEAC assay is shown in (b). The decolourisation of the radical-solution was detected spectrophotometrically at 734 nm (mean values ± SD, *n* = 3, ^*^ *P* < 0.05 versus control value, Student's *t*-test).

To investigate if TSG may exert antioxidative effects in* C. elegans*, we have analysed the radical scavenging capacity of this compound in the trolox equivalent antioxidative capacity (TEAC) assay. In this cell-free assay, the potency of a substance to reduce and thereby decolourise a green radical is detected. We have compared the antioxidative capacity of TSG with the structural analogue resveratrol and quercetin, a major flavonoid with a well-known antioxidant capacity. TSG possesses a strong antioxidative capacity in this system (Figure 1(b)). Even at the lowest concentration analysed (5 *μ*M), TSG showed a strong antioxidative effect; about 30% of the ABTS radical was reduced. The compound reduced the ABTS radical even more efficiently than resveratrol over the complete concentration range. Up to a concentration of 15 *μ*M, TSG shows a higher antioxidative capacity than trolox, the synthetic vitamin E derivative that was used as a reference substance. Out of the compounds analysed, only the flavonoid quercetin showed stronger antioxidative effects over the complete concentration range. The superior antioxidative capacity of quercetin in the TEAC assay can be explained by differences in the molecular structure of this compound compared to resveratrol and TSG. In contrast to the both stilbene derivatives, the flavonoid possesses redox-active moieties which facilitate the reduction of the ABTS radical. These groups are, for example, the catechol group in ring B in combination with the 3-OH-group in ring C. These groups are able to donate electrons to the ABTS radical forming stable semiquinone radicals and quinoid structures. In the case of the stilbene derivatives, the stabilisation of the oxidized form is not favoured as in case of quercetin.

**Figure 2:**
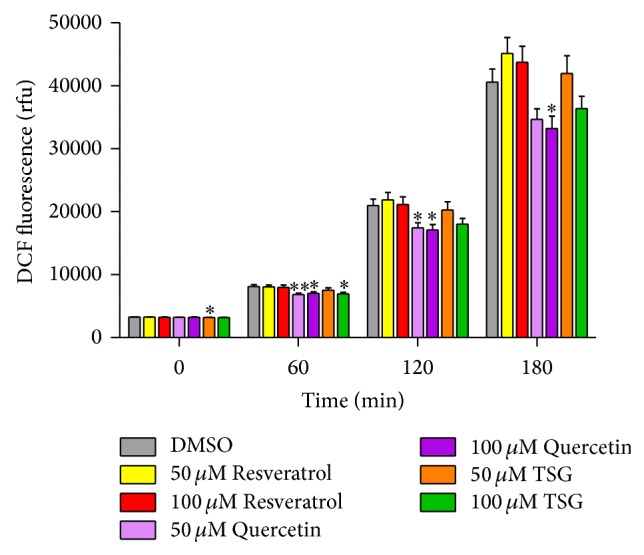
Modulation of ROS accumulation in* C. elegans* by TSG. Modulation of ROS accumulation in wild type nematodes at 37°C after incubation with TSG, quercetin, and resveratrol (50, 100 *μ*M). The fluorescence intensity of DCF (rfu) was used as a marker for intracellular ROS; mean ± SEM, 5–7 independent experiments; at least 80 animals per concentration were analysed; ^*^ *P* < 0.05 versus* DMSO value*, one-way ANOVA with Dunnett's post hoc test.

Next, we investigated the antioxidative effects of TSG in the nematode* C. elegans*. To analyse the antioxidative potential* in vivo*, the DCF assay was used. DCF is a probe that becomes highly fluorescent after oxidation; therefore the DCF fluorescence was taken as a marker for the formation of reactive oxygen species within the organism. We induced the formation of reactive oxygen species in* C. elegans* by application of thermal stress (37°C). As shown in Figure 2, the amount of fluorescent DCF increases over time after the onset of stress conditions. After 180 minutes, the DCF fluorescence of DMSO-treated nematodes was approximately 12.4-fold higher compared to the DMSO value at *t* = 0 (3276 ± 22 rfu → 40600 ± 2055 rfu). Treatment of the nematodes for 48 h with 100 *μ*M TSG reduced the stress-induced increase in ROS: after 180 min, the rfu-value was 36378 ± 1926. The experiments performed with resveratrol showed no significant modulation of the DCF fluorescence. However, an incubation with the well-known antioxidant flavonoid quercetin caused a significant reduction of DCF fluorescence at 100 *μ*M.

Antioxidative effects of resveratrol are extensively described in the literature: Jang and Surh [36] described protective effects of resveratrol on hydrogen peroxide-induced apoptosis in rat pheochromocytoma (PC12) cells; also in H4IIE rat hepatoma cells antioxidative effects were reported in the DCF assay [37]. In a more recent paper, Vanaja et al. [38] described an improvement of the antioxidative properties of resveratrol after loading into liposomes. The antioxidative properties of resveratrol are further reviewed by, for example, Farghali et al. [39]. Concerning the antioxidative effects of TSG, less information is available: Kim et al. [40] reported protective effects of an extract of* Polygonum multiflorum* against oxidative toxicity in HT22 hippocampal cells without showing active components of the extract. Chen et al. [15] identified antioxidative components of* Polygonum multiflorum* using the 2,2-diphenyl-1-picrylhydrazyl (DPPH) assay (similar to TEAC assay). They reported a strong antioxidative capacity by only three compounds: gallic acid, catechin, and TSG. Further antioxidative effects are reported by Zhang et al. [41] demonstrating protective effects of TSG against hydrogen peroxide-induced dysfunction and oxidative stress in osteoblastic MC3T3-E1 cells; Tao et al. [21] report a protective effect of TSG on 6-OHDA-induced apoptosis in PC12 cells and Li et al. [24] demonstrate that TSG attenuates MPP^+^-induced apoptosis in PC12 cells by inhibiting ROS generation.

### 3.2. Modulation of Antioxidative Enzyme Expression by TSG

Polyphenols like TSG may protect against oxidative stress either by directly scavenging radicals or by indirectly increasing the stress resistance of the organism, for example, by induction of antioxidative enzymes. We have investigated the effects of TSG on the induction of glutathione-S-transferase-4 (GST-4) and superoxide dismutase-3 (SOD-3), two enzymes that are known to be inducible by oxidative stress.

**Figure 3:**
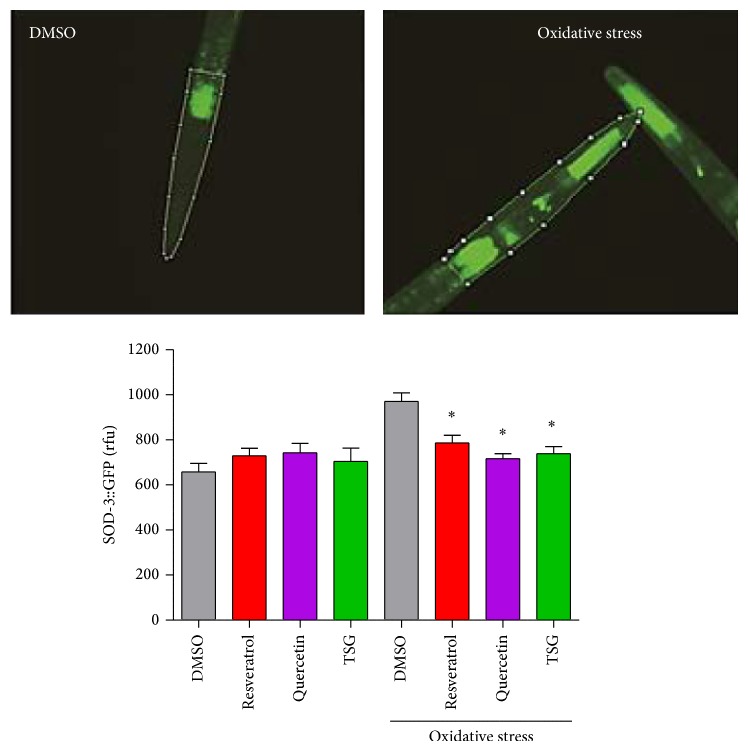
Modulation of SOD-3 expression by TSG. The images show GFP fluorescence of the head and anterior part of the intestine of CF1553 transgenic nematodes pretreated with 100 *μ*M of the compounds without (DMSO) or with oxidative stress (150 *μ*M juglone; 3 h). Data represent mean values ± SEM, *n* = 3, ^*^ *P* < 0.05 versus corresponding DMSO value, one-way ANOVA with Dunnett's post hoc test. At least 10 animals per group and experiment were analysed.

To analyse the induction of SOD-3::GFP expression in* C. elegans*, we used the transgenic strain CF1553 (*muIs84 [pAD76(sod-3::gfp)]*). Treatment with TSG, resveratrol, or quercetin showed no significant influence on the expression of SOD-3 under basal conditions (Figure 3). Under stress conditions (150 *μ*M of the redox-cycler juglone), SOD-3 expression is induced in* C. elegans*. Compared to the GFP fluorescence of nematodes under basal conditions (657.3 rfu), the fluorescence increased to 970.8 rfu under conditions of oxidative stress. This result shows that the redox-state in the nematode after application of juglone was shifted to the prooxidative state; a relatively high induction of the antioxidative enzyme SOD-3 was needed to compensate this prooxidative condition. Treatment of the nematodes with 100 *μ*M TSG starting 48 h prior to the application of juglone results in a significantly reduced induction of SOD-3 expression (704.1 rfu → 738.7 rfu). This may be interpreted as a reduction of the juglone-induced prooxidative status of the nematode that in consequence results in a lower expression of SOD-3. The effect of resveratrol was comparable to the effect of TSG; the highest modulation was caused by quercetin.

**Figure 4:**
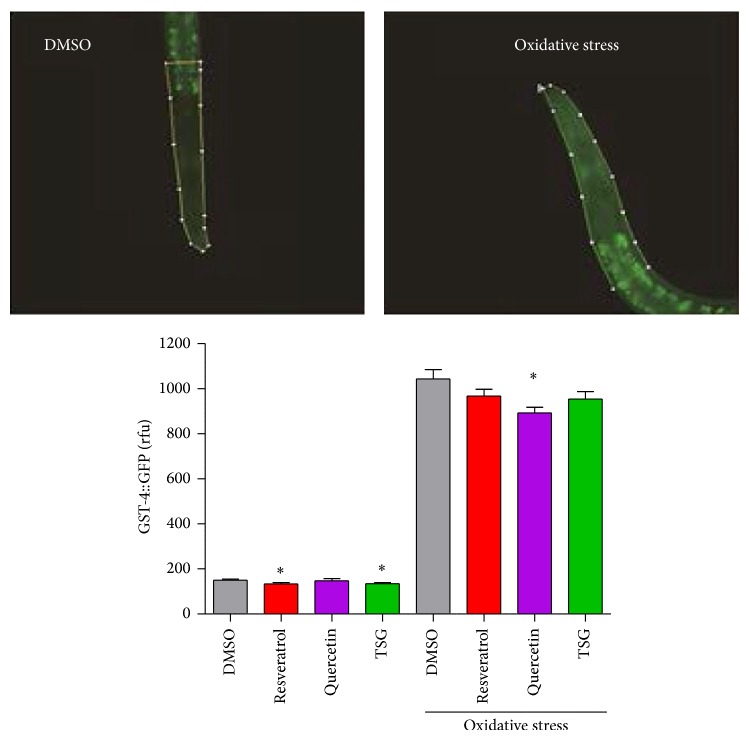
Modulation of GST-4 expression by TSG. The images show GFP fluorescence of the head and the anterior part of the intestine of CL2166 transgenic nematodes pretreated with 100 *μ*M of the compounds followed by an incubation under physiological conditions or under conditions of oxidative stress (150 *μ*M Juglone; 3 h). Data represent mean values ± SEM, *n* = 3, ^*^ *P* < 0.05 versus corresponding DMSO value, one-way ANOVA with Dunnett's post hoc test. At least 10 animals per group and experiment were analysed.

Next, we analysed the effects of TSG on the induction of GST-4, an enzyme that is thought to be involved in the defence against conditions of stress due to the induction via SKN-1. Similar to the SOD-3 reporter gene experiment, we used a transgenic strain (CL2166* dvIs19 [pAF15(gst-4::gfp::NLS)]*) that expresses GFP under control of the* gst-4* promoter (Figure 4). Comparable to the expression of SOD-3, the expression of GST-4 is strongly increased under stress conditions. A 6.9-fold increase in the GFP fluorescence was detected after application of juglone (150.62 ± 5 rfu → 1043.16 ± 42 rfu). In contrast to the effect observed with the transgenic strain CF1553 (= SOD-3 reporter), TSG and resveratrol only slightly modulated the induction of GST-4; only a tendency can be suggested. Quercetin was the only compound that significantly diminished the induction of GST-4 after application of oxidative stress. The modulation of SOD-3 and GST-4 by the compounds can be explained in two ways. On the one hand, the direct antioxidative potential of the compounds reduces the oxidative stress which is generated by the redox-cycler juglone. The reduction in oxidative stress consequently reduces the amount of antioxidative enzymes which are induced. On the other hand, the reduction of SOD-3 induction may also be due to an indirect antioxidative effect of the compounds by specific activation of the antioxidative response system of the nematode.

Robb and Stuart [42] reported a significantly increased MnSOD expression by resveratrol in mouse C2C12 and primary myoblasts. Khan et al. [43] reported that treatment with 25 *μ*M resveratrol significantly increased SOD activity in PC-3, HepG2, and MCF-7 cells, but not in HEK293T cells. Kavas et al. [44] reported an increase of SOD activity in male Wistar rats by resveratrol (20 mg/kg in drinking water for six weeks). Li et al. [45] reported that resveratrol treatment significantly increased the mRNA expression of GSTA1 in a time-dependent manner. On the other hand, Jiang et al. [46] showed that resveratrol attenuates early diabetic nephropathy by downregulating glutathione S-transferase Mu in diabetic rats. In contrast to the vast information about resveratrol, to our best knowledge, no information about TSG and modulation of antioxidative enzymes is available.

### 3.3. Modulation of Stress Resistance by TSG

**Figure 5:**
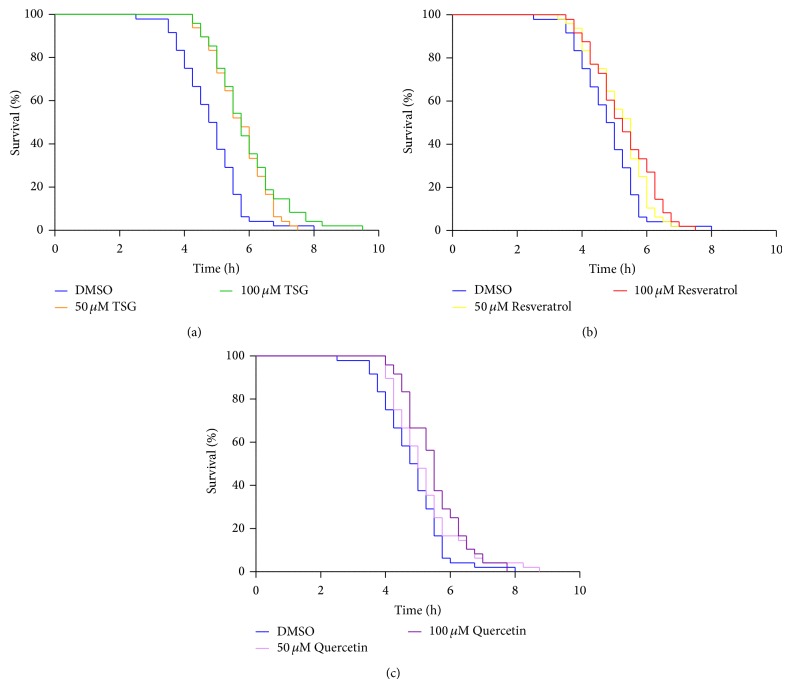
Increased resistance against lethal heat-stress by treatment with TSG. TSG treatment increases the resistance against thermal stress at 50 *μ*M and 100 *μ*M (a). Resveratrol treatment (b) and quercetin treatment effectively increased the survival only at 100 *μ*M. Survival curves represent the data of 4 independent experiments with a total of 48 animals per group (Kaplan-Meier survival analysis); corresponding data are summarised in Table 1.

Since TSG reduces the abundance and induction of reactive oxygen species in* C. elegans*, we investigated if the stilbene derivative also mediates a resistance against thermal stress (37°C) which has been shown to be lethal in* C. elegans*. We have analysed the effects of TSG, resveratrol, and quercetin on the tolerance of* C. elegans* against thermal stress using the semiautomated SYTOX Green assay (Figure 5). The mean and median survival time of DMSO-treated nematodes were determined as 4.82 ± 0.14 h and 4.75 ± 0.17 h, respectively. TSG strongly increases the resistance against thermal stress: 50 *μ*M TSG induces a 22% increase in the mean survival time. The mean and median survival time of TSG-treated nematodes were determined as 5.89 ± 0.11 h and 6.00 ± 0.1 h, respectively. In case of stress resistance, TSG was shown to be the most active compound analysed (Table 1). Combined with the experiments shown before, this result clearly indicates that TSG has a high potential to protect* C. elegans* against stress conditions. Chen et al. [47] reported that resveratrol alleviated juglone-induced lethal oxidative stress and significantly prolonged the survival time of* C. elegans* under conditions of acute oxidative damage. However, no information is available about effects of TSG on stress resistance.

### 3.4. Prolongation of Life Span by TSG

With increasing age, highly oxidised and cross-linked proteins accumulate in the intestine of the nematode. These modified molecules form insoluble, autofluorescent ageing pigments, for example, lipofuscin. We investigated the effect of TSG, resveratrol, and quercetin on this phenomenon (i) to verify the antioxidative effects of the compounds in the nematode and (ii) to estimate effects of these compounds on the ageing process in* C. elegans*.

**Figure 6:**
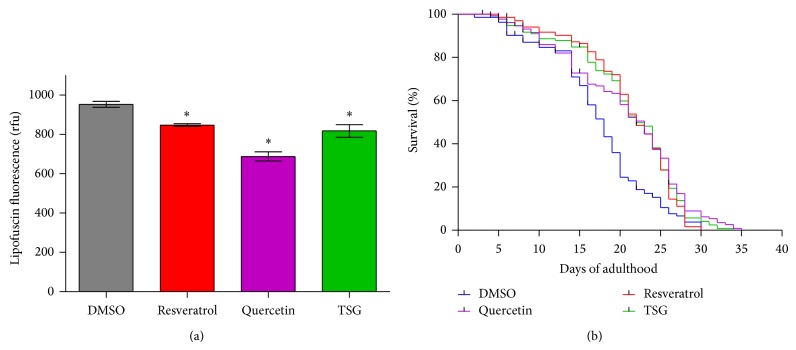
Prolongation of life span by treatment with TSG. (a) Pretreatment with 100 *μ*M of TSG, resveratrol, and quercetin reduced the accumulation of the ageing marker lipofuscin (mean values ± SEM, *n* = 3, ^*^ *P* < 0.05 versus control value, Student's *t*-test). 20 animals per group and experiment were analysed. (b) All compounds (100 *μ*M) induced a prolongevity effect during the complete adult life (Kaplan-Meier survival analysis of three independent experiments with at least 30 animals per group/experiment; survival data are summarised in Table 2).

Treatment with TSG reduces the intestinal lipofuscin accumulation in* C. elegans* by 20 ± 1% compared to the solvent control (DMSO: 952 ± 19 rfu; TSG 817 ± 16 rfu). Resveratrol showed a slightly weaker decrease in lipofuscin fluorescence (846.8 rfu), while quercetin again showed the strongest reduction in this parameter. Incubation with 100 *μ*M quercetin reduces the lipofuscin fluorescence by 27.85% (686.9 ± 14 rfu). These results show that TSG is capable of decreasing lipofuscin accumulation in* C. elegans* (Figure 6(a)).

Since it is known that a reduction of lipofuscin mostly correlates with an increase in life span of* C. elegans*, we investigated the effect of TSG, resveratrol, and quercetin on the life span of* C. elegans*. All three compounds exerted similar effects (Figure 6(b)). The mean life span of DMSO-treated nematodes was 17.4 ± 0.56 days; in case of TSG, resveratrol, and quercetin, the mean life span was 21.1 ± 0.58 d, 20.7 ± 0.67 d, and 21.3 ± 0.5 d, respectively (Table 2). The median life span of DMSO-treated nematodes was 18.0 ± 0.61 days; in case of TSG, resveratrol, and quercetin, the median life span was 22.0 ± 0.81 d, 22.0 ± 0.71 d, and 23.0 ± 1.01 d, respectively.

From the results of the DCF assay we can conclude that the radical scavenging effects of the compounds cannot be the only explanation for their positive effects on the life span of* C. elegans*. The antioxidative capacity of resveratrol was negligible in comparison to TSG and quercetin, but the effect on the life span of the nematode is comparable to the other two compounds.

**Figure 7:**
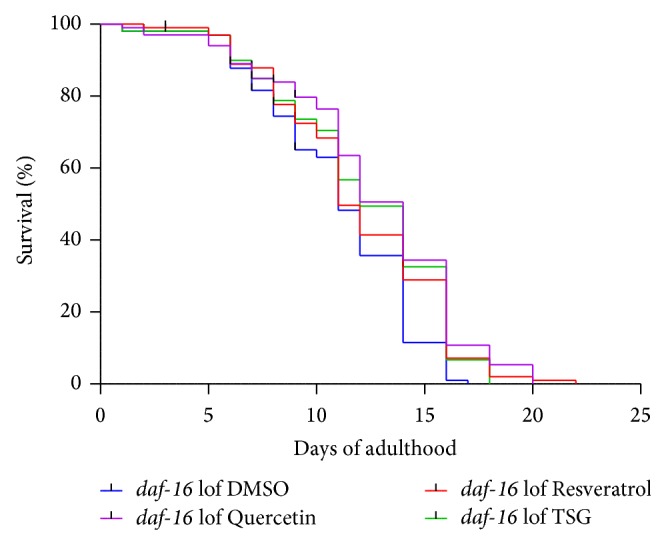
The TSG-mediated prolongation of life span is independent of DAF-16. Treatment with the compounds (100 *μ*M) during the complete adult life induced a prolongevity effect in the* daf-16* loss-of-function mutant strain CF1038 (Kaplan-Meier survival analysis of two independent experiments with at least 30 animals per group/experiment; survival data are summarised in Table 2).

To clarify this point, we analysed if the compounds may modulate intracellular pathways to prolong the life span of* C. elegans*. Therefore we used a mutant strain that contains a deletion in the* daf-16* gene leading to a loss-of-function of the corresponding protein. This transcription factor (FoxO homologue in* C. elegans*) has a crucial function in the regulation of ageing. By using a mutant strain defective in this pathway, we analysed if this factor is involved in the life span-prolonging effect of TSG. As we see in Figure 7, the life span extending effect of TSG, quercetin, and resveratrol was not abolished. We conclude that this pathway is not necessary to mediate the effects of TSG, quercetin, and resveratrol.

Resveratrol has been reported to be beneficial in cases of ageing-related cardiovascular and neurodegenerative diseases. However, previous studies on the longevity promoting effect of resveratrol have been partly inconclusive. Upadhyay et al. [48] reported an increase of life span after treatment with resveratrol (100 *μ*M). Zarse et al. [49] reported that resveratrol significantly extends* C. elegans* life span already at a concentration of 5 *μ*M by 3.6% (mean life span) and 3.4% (maximum life span). On the other hand, Chen et al. [47] observed no extension of the normal life span of* C. elegans* in either liquid or solid growth media containing different concentrations of resveratrol. Bass et al. [50] also analysed effects of resveratrol in* C. elegans* (wild type and sir-2.1 mutant nematodes) but results were variable. Resveratrol treatment results in slight increases in life span in some trials but not others (wild type and sir-2.1 mutant animals). As an explanation for the different effects there may be variations from one study to another concerning the delivery of the compounds to the nematodes. The use of liquid or solid growth media containing different concentrations of resveratrol makes it also difficult to compare results between studies. In our study, we confirm results of Upadhyay et al. [48] showing an extension of life span after incubation with resveratrol. Furthermore, we were the first to show that also application of TSG results in a prolongation of life span comparable to resveratrol. This finding may be a hint for the active component in the* Polygonum multiflorum* extract, which is traditionally used as an anti-ageing drug. It has to be mentioned that no adverse effects of the compounds (TSG, resveratrol, and quercetin) were detectable in the experimental assays up to a concentration of 100 *μ*M. This is important since it was reported that other natural compounds cause toxic effects to the nematode: Mukai et al. [51] reported that a gallate of tannin isolated from the tea plant* Camellia sinensis* L. is toxic to* C. elegans* (LC_50_: 49 *μ*M).

## 4. Conclusion

2,3,5,4′-Tetrahydroxystilbene-2-O-*β*-D-glucoside (TSG) is the main bioactive component of* Polygonum multiflorum*, a plant which is traditionally used as an anti-ageing agent in many East Asian countries. This compound causes antioxidative effects in* C. elegans*, alleviates the accumulation of lipofuscin, and prolongs the mean life span by 23.5% independently of DAF-16. Furthermore, the stress resistance of the nematode is strongly enhanced by this compound. In addition to direct antioxidative effects of TSG, this compound also causes indirect antioxidative effects in* C. elegans* via modulation of SOD-3 and GST-4. Our results strongly confirm the potential of TSG to be used as a pharmaceutical anti-ageing drug.

## Figures and Tables

**Table 1:** Summary of the heat-stress survival data depicted in Figure 5.

Adult survival [*h* ± SEM] at 37°C				
Treatment	Mean	Median	*n*	*P* versus DMSO (log-rank)
DMSO	4.82 ± 0.14	4.75 ± 0.17	48	
Quercetin (50 *µ*M)	5.22 ± 0.15	5.00 ± 0.16	48	0.10
Quercetin (100 *µ*M)	5.50 ± 0.13	5.50 ± 0.09	48	<0.001
Resveratrol (50 *µ*M)	5.20 ± 0.13	5.25 ± 0.16	48	0.05
Resveratrol (100 *µ*M)	5.29 ± 0.14	5.25 ± 0.24	48	<0.05
TSG (50 *μ*M)	5.89 ± 0.11	6.00 ± 0.10	48	<0.001
TSG (100 *μ*M)	5.94 ± 0.17	5.75 ± 0.12	48	<0.001

**Table 2:** Summary of the life span analyses of wild type nematodes (Figure 6(b)) and *daf-16* loss-of-function mutants (strain CF1038) (Figure 7).

Wild type	Adult life span [*d* ± SEM]			
Treatment	Mean	Median	*n* (censored)	*P* versus DMSO (log-rank)
DMSO	17.39 ± 0.56	18.00 ± 0.61	121 (14)	
Quercetin (100 *µ*M)	20.68 ± 0.67	23.00 ± 1.00	118 (17)	<0.001
Resveratrol (100 *µ*M)	21.31 ± 0.50	22.00 ± 0.71	129 (6)	<0.001
TSG (100 *μ*M)	21.14 ± 0.58	22.00 ± 0.81	128 (7)	<0.001

*daf-16(mu86) *	Adult life span [*d* ± SEM]			
Treatment	Mean	Median	*n* (censored)	*P* versus DMSO (log-rank)

DMSO	11.05 ± 0.35	11.00 ± 0.37	96 (4)	
Quercetin (100 *µ*M)	12.64 ± 0.43	14.00 ± 0.51	94 (6)	<0.001
Resveratrol (100 *µ*M)	12.01 ± 0.40	11.00 ± 0.38	97 (3)	0.018
TSG (100 *μ*M)	12.21 ± 0.41	12.00 ± 0.63	94 (6)	0.02

## Abbreviations

DCF: 2′,7′-Dichlorofluorescein
DMSO: Dimethylsulfoxid
GST: Glutathione-S-transferase
rfu: Relative fluorescence unit
ROS: Reactive oxygen species
SOD: Superoxide dismutase
TCM: Traditional Chinese medicine
TEAC: Trolox equivalent antioxidative capacity assay
TSG: 2,3,5,4′-Tetrahydroxystilbene-2-O-*β*-D-glucoside.
